# Correction to “Impact analysis of heart failure across European countries: an ESC‐HFA position paper” and “Echocardiographically defined haemodynamic categorization predicts prognosis in ambulatory heart failure patients treated with sacubitril/valsartan”

**DOI:** 10.1002/ehf2.14672

**Published:** 2024-01-11

Massimo Piepoli's affiliations was incorrect.

It should have read:

- Department of Biomedical Science for Health, University of Milan, Via Festa del Perdono 7, 20122 Milan, Italy
- Clinical Cardiology, IRCCS Policlinico San Donato, Via Morandi 30, 20097 San Donato Milanese, Milan, Italy

The affected articles are listed in the reference list.^1^, ^2^

We apologize for these errors.
